# Innovative Research Approaches to Cope with Water Security in Africa

**DOI:** 10.1002/ieam.4337

**Published:** 2020-10-06

**Authors:** Angel de Miguel, Jochen Froebrich, Atef Jaouani, Yasmine Souissi, Amgad Elmahdi, Javier Mateo‐Sagasta, Mohamed Al‐Hamdi, Dario Frascari

**Affiliations:** ^1^ Water and Food Team, Wageningen Environmental and Research Wageningen the Netherlands; ^2^ Laboratory of Microorganisms and Active Biomolecules, Faculty of Sciences of Tunis, University de Tunis El Manar El Manar Tunisia; ^3^ University of Manouba, ISBST Ariana Tunisia; ^4^ International Water Management Institute‐IWMI Colombo Sri Lanka; ^5^ Food and Agriculture Organization of the United Nations (FAO), Regional Office for the Near East and North Africa Region (RNE) Cairo Egypt; ^6^ Department of Civil Chemical, Environmental and Materials Engineering, University of Bologna Bologna Italy

**Keywords:** Water security, Africa, Treated wastewater, Drinking water, Water management

## Abstract

To achieve a water‐secure world, water management should be approached from a multidimensional and integrative perspective, addressing the water‐related issues of health, household supply, economics, the environment, and resilience to water‐related and climate change hazards. Although water security has significantly improved since 2000 in Africa, there are still vast inequalities in access to water suitable in terms of quantity and quality, especially in rural areas. To achieve water‐related sustainable development of African economies, a broad scope of innovative technological and management solutions is required, involving governments, research institutions, private sector parties, and civil society. This special series, composed of 8 papers, illustrates a selection of the most relevant results achieved by the 7 research projects selected and financed by the European Union under 2 dedicated Horizon 2020 calls in 2015: Water‐5b‐2015 “A coordination platform” and Water‐5c‐2015 “Development of water supply and sanitation technology, systems and tools, and/or methodologies.” The innovations presented in this special series include both technological advancements and w'ater management approaches, given that the development of water‐related technologies in developing countries needs to be integrated into water management strategies and economic instruments. This special series aims to help policy makers take informed decisions on how to implement innovative approaches to increase water security in African countries. *Integr Environ Assess Manag* 2020;16:853–855. © 2020 The Authors. *Integrated Environmental Assessment and Management* published by Wiley Periodicals LLC on behalf of Society of Environmental Toxicology & Chemistry (SETAC)

## KEY POINTS

- To achieve a more water secure world, water management should be approached from a multidimensional and integrative perspective.
- This special series aims to help policy makers take informed decisions on how to implement innovative approaches to increase water security in African countries.

## INTRODUCTION

Water security accounts for the lack of risk of communities to suffer water‐related issues in terms of both quantity and quality, as well as the capacity to cope with those threats (Cook and Bakker 2012). To achieve a water‐secure world, water management should be approached from a multidimensional and integrative perspective, addressing the water‐related issues of health, household supply, economics, the environment, and resilience to water‐related and climate change hazards (ADB 2016). In a context of population and economic growth, social fragility, land use changes, and climate change, closing the gaps between water availability in terms of quantity and quality and demand is a priority issue to cope with.

According to the World Health Organization (WHO 2019), one‐third of the global population does not have access to safe drinking water and more than 4 billion people do not have safely managed sanitation services. Developing countries are particularly vulnerable to water‐related threats. Although water security has significantly improved since 2000 thanks to the higher access of people to safe water and sanitation services, there are still vast inequalities in access to water of sufficient quantity and quality in African countries, especially in rural areas. Water stress is also a major concern in many places of Africa because the total water withdrawals are close to or higher than the available renewable water supplies. According to the Aquaeduct database (Hofste et al. 2019), 15 out of the 54 African countries present medium to extremely high water stress. A water‐related sustainable development of African economies is essential in order to guarantee food security, health, societal well‐being, and economic growth. To achieve this goal, a broad scope of innovative technological and management solutions is required, involving governments, research institutions, private sector parties, and civil society. Water security in Africa should be addressed with interventions at different scales, from simple hands‐on improvements at household scale up to complex multinational and multistakeholder approaches.

In order to better tackle water‐related challenges in Africa, in 2015 the European Union launched 2 dedicated Horizon 2020 calls: Water‐5b‐2015 “A coordination platform” and Water‐5c‐2015 “Development of water supply and sanitation technology, systems and tools, and/or methodologies.” This special series illustrates a selection of the most relevant results achieved by the 7 research projects selected and financed by the European Union in the context of these 2 calls, displaying how different types of innovative solutions—from local interventions at household level to water security approaches at multinational scale—can increase the water‐related resilience of African citizens. The innovations presented in this special series include both technological advancements and water management approaches, given that the development of water‐related technologies in developing countries needs to be integrated into the definition and implementation of water management strategies and economic instruments (Frascari et al. 2018). The technological and management innovations presented in this special series aim to contribute to the achievement of the United Nations Sustainable Development Goals (SDGs), with particular focus on SDG 6 “Clean water and sanitation” and—given the close nexus between water and food security—SDG 2 “Zero hunger.”

## CONTENT OF THE PAPERS

The first 2 papers of this series (Idini et al. this issue; Morse et al. this issue) focus on the development and assessment of technologies to guarantee access of rural communities to safe drinking water at the household level. Both papers include not only evaluation of technical aspects of the proposed technologies but also analysis of the potential barriers that hinder their adoption, such as a social acceptability, regulations, and robustness. Idini et al. (this issue) investigated the effectiveness of an innovative defluorination device and the willingness of rural communities in the East African Rift Valley, where natural fluoride contamination of drinking water is widespread, to buy and use the proposed device. The device, powered by a car battery, produces defluorinated water at an affordable cost (US$0.03/L), but the low level of awareness about fluorosis diseases in the population makes its widespread adoption challenging. Morse et al. (this issue) explored how to break barriers to the use of well‐proven solar water disinfection technology (SODIS) among the rural population in Malawi. They applied a transdisciplinary behavior‐centered design in order to develop an appropriate and acceptable system and a context‐specific intervention delivery program. A large number of surveys of SODIS users allowed the identification of specific contextual issues and behavioral determinants that guided the development and delivery mechanism of the SODIS toolkit.

The second group of papers focuses on the use of treated wastewater for irrigation, a practice that can significantly contribute to increased water and food security in Africa. Although the use of safely managed sanitation services in African countries is still far from acceptable, the number of people connected to a sewer has increased in the last 10 y. This is especially true for the urban population of North African countries, where 77% of the population is connected to a sewer and 38% to a treatment facility (WHO 2019). Among other benefits, treated wastewater (TWW) reuse allows farmers to have a reliable source of water and nutrients, which decreases their vulnerability to climate variability and reduces their dependence on external fertilizers. Thanks to recent technological developments, high‐quality TWW can be produced, drastically reducing the potential risk for users and consumers. However, some technologies are expensive and difficult to implement in a rural context. Thus, innovative approaches and technologies more adapted to the African context are required, addressing not only the technological barriers to TWW reuse but also the social and economic ones.

Oertlé et al. (this issue) developed a comprehensive assessment for TWW reuse, based on the development of a decision‐support tool (DST) that can 1) develop combinations of wastewater treatment technologies for the production of irrigation‐quality water from different types of wastewater, and 2) estimate the life cycle costs of the proposed treatment technologies. The DST was tested in the context of 3 North African countries (Egypt, Morocco, and Tunisia) showing that, even though these countries have a high potential for TWW reuse, the practice is poorly implemented due to numerous barriers related to water management, water and TWW pricing, subsidies, implementation of monitoring and reporting systems, and legal obstacles to TWW reuse. Focusing on a more sectoral scale, Oubelkacem et al. (this issue) evaluated the economic feasibility of irrigation and nutrient management with TWW in the citrus sector in the Souss Massa region, Morocco. Considering the effects of TWW reuse on crop yields and on water and fertilizer requirements, a mathematical nonlinear optimization model was used to identify the optimal allocation of land, fresh water, and TWW. The paper, which includes the assessment of the impact of such optimization on the economic performance of the citrus sector in Morocco, concludes that TWW reuse must be significantly subsidized in order to make it a convenient alternative for irrigation in Morocco.

Dragonetti et al. (this issue) developed a safe irrigation management (SIM) predictive model, aiming to improve current irrigation practices, accounting for water quality, soil characteristics, and crop yield at farm level. The model, adapted to TWW reuse, was tested on a citrus farm in Souss‐Massa, Morocco, showing that—in comparison with the current irrigation strategy typically adopted by farmers—the SIM strategy allows attainment of a significant reduction of water and fertilizer consumption, and consequently of fertilizer leaching and groundwater pollution. With a more technology‐oriented approach, Pinelli et al. (this issue) assessed the technical, environmental, and financial feasibility of upgrading the existing drainage canals in the Nile Delta (Egypt) to treat the effluents from drainage and municipal wastewater in order to produce water that is reusable in agriculture. Two technologies were tested, an in‐stream constructed wetland and a canalized facultative lagoon. The upgrade of drainage canals to wastewater treatment facilities appears a promising option in the Nile Delta, thanks to the high pollutant removal performances, low cost, and negligible environmental burden. The higher quality of the irrigation water could induce downstream farmers to switch from nonfood crops, such as cotton, to more profitable food crops, such as rice.

The last 2 papers of this series deal with TWW reuse in aquaculture systems. Aquaculture is gaining prominence in Africa as a source of food and income, especially in the Great Lakes area. However, the vast majority of East African aquaculture is open‐pond based, requiring large amounts of water and causing pollution issues. Both papers focus on the implementation and assessment of a modern and sustainable aquaculture technology adapted to the local context of Kisumu, Kenya. The hatchery, powered by a photovoltaic system, consists of a recirculating aquaculture system (RAS) coupled to a membrane bioreactor (MBR). Clough et al. (this issue) report the main results from the RAS, which proved able to reduce by 90% to 95% the consumption of lake water thanks to the recirculation technology. Gukelberger et al. (this issue) focus on the technical evaluation of the MBR, capable of producing high‐quality water from Kisumu's municipal wastewater. This TWW can be used to compensate evaporation losses in the hatchery and also to irrigate a variety of local vegetables. The MBR technology also shows promising applications in the fish processing industry, reducing freshwater consumption and minimizing the discharge of highly polluted effluents in the lakes. The final aim is to reduce the pressure on the Great Lakes, which are in serious risk of collapse due to overfishing and severe pollution.

The combination of innovative technological and management solutions presented in this special series, ranging from drinking water production to TWW reuse for irrigation and aquaculture, is expected to help policy makers take informed decisions on how to implement innovative approaches to increase water security in African countries.

## Disclaimer

The authors declare no conflicts of interest.

## Data Availability

No additional data set was used for this introductory paper.
